# Supplementary material for: Quantifying electron-transfer in liquid-solid contact electrification and the formation of electric double-layer
Source: Nat Commun. 2020 Jan 21;11:399. doi: 10.1038/s41467-019-14278-9 (PMC6972942; doi:10.1038/s41467-019-14278-9)
Supplement: Supplementary file 1 — Supplementary Information [file 41467_2019_14278_MOESM1_ESM.pdf]

Supplementary Information

Quantifying electron-transfer in liquid-solid contact electrification and the formation of electric double-layer

Lin *et al.*

Supplementary Figures

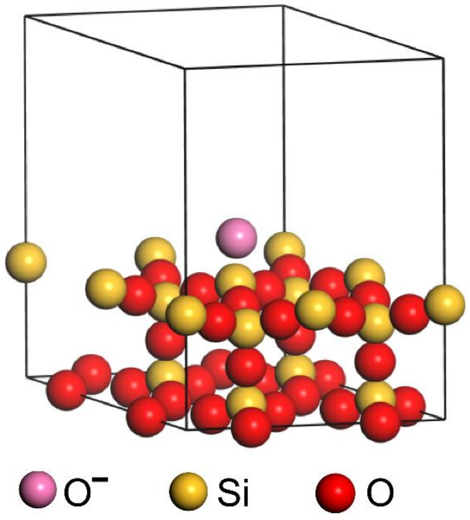

Supplementary Figure 1. The initial structure of the SiO_2 surface with an O^- ion.

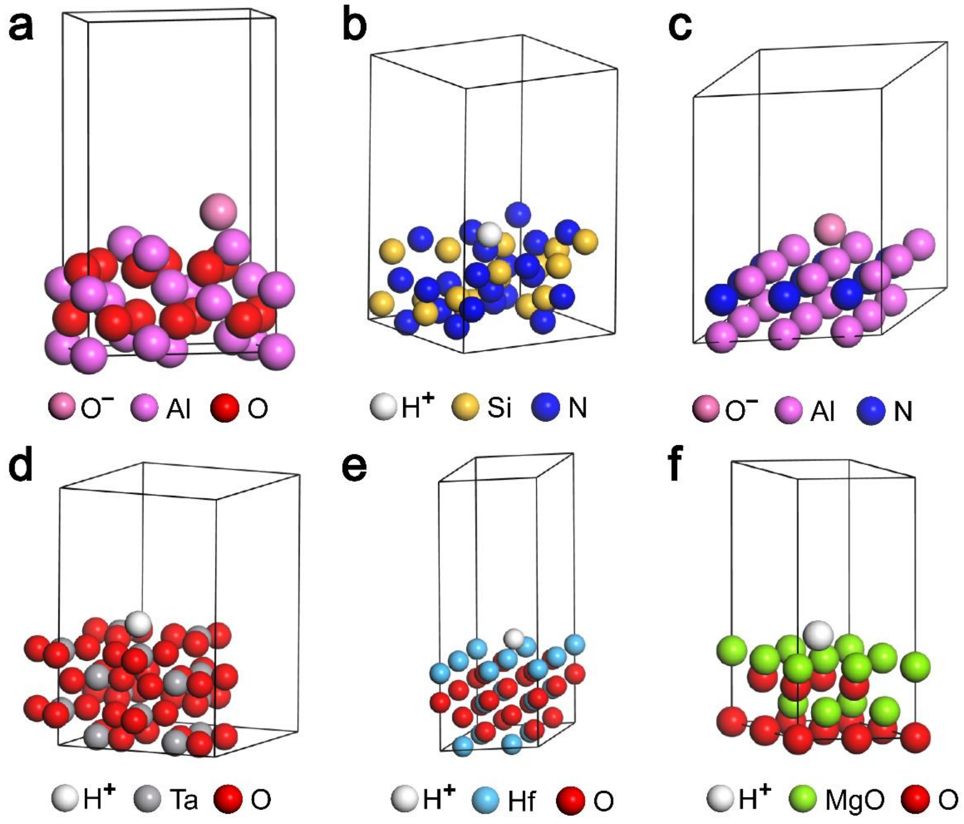

Supplementary Figure 2. The structures of the models. The initial structure of the (a) Al_2O_3, (b) Si_3N_4, (c) AlN, (d) Ta_2O_5, (e) HfO_2 and (f) MgO surfaces with an O^- ion (negative charge) or H^+ ion (positive charge).

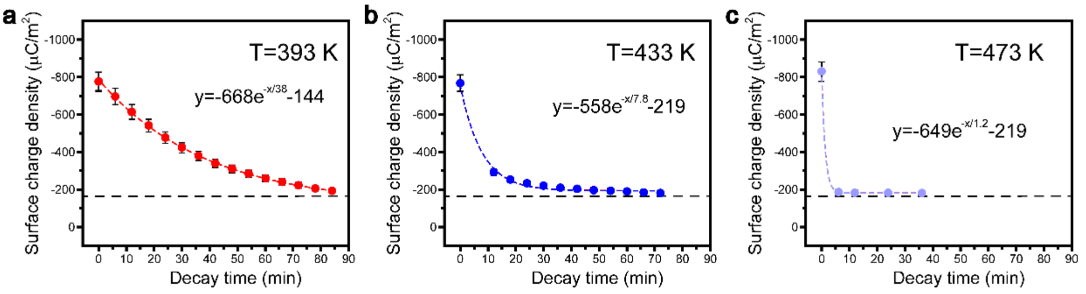

Supplementary Figure 3. The fitting of the charge decay curves. When the temperature is (a) 393 K, (b) 433 K and (c) 473 K. (Error bar are defined as s. d.).

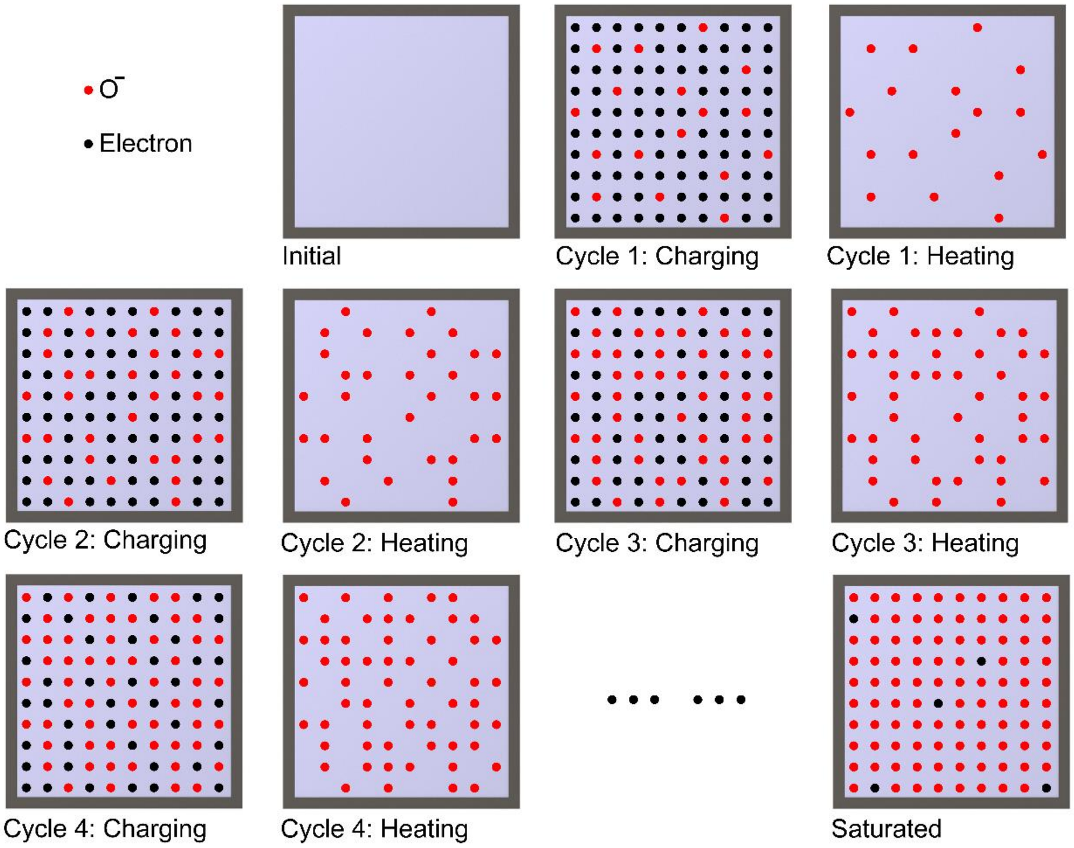

Supplementary Figure 4. Illustration of O^- ions and electrons on SiO_2 surface in the cycle tests.

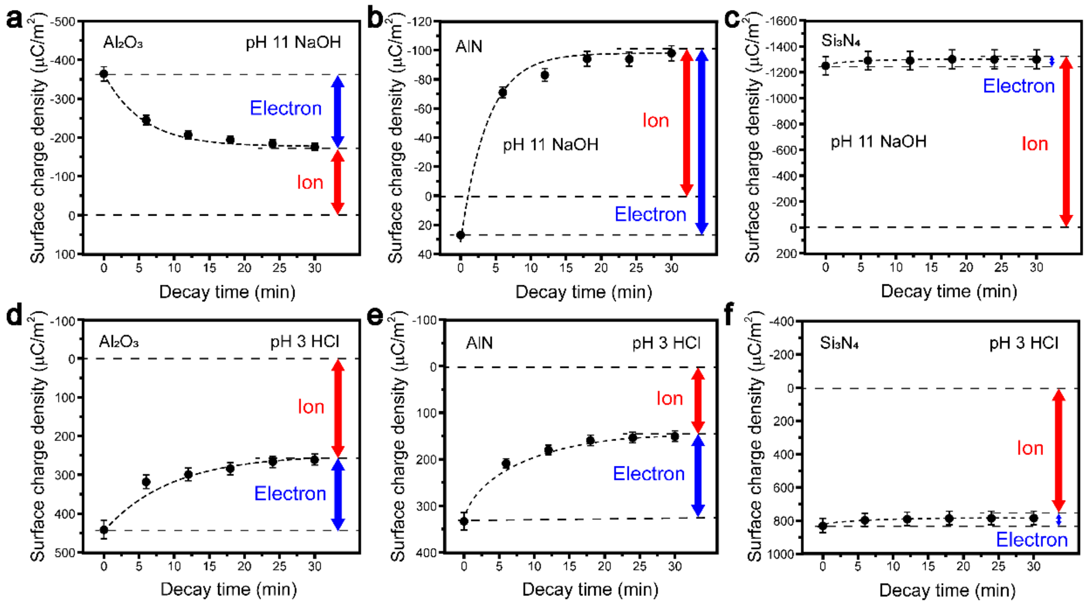

Supplementary Figure 5. The effects of pH conditions of the solution on the charge decays. The decay of the triboelectric charge (induced by contacting with pH 3 HCl solution) on the (a) Al_2O_3, (b) AlN, (c) Si_3N_4 surfaces under 433 K. The decay of the triboelectric charge (induced by contacting with pH 11 NaOH solution) on the (d) Al_2O_3, (e) AlN, (f) Si_3N_4 surfaces under 433 K. (Error bar are defined as s. d.).

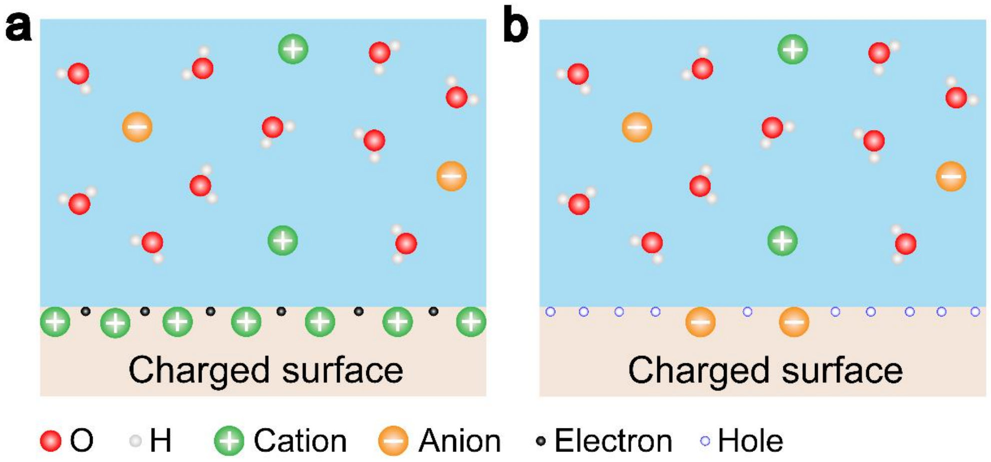

Supplementary Figure 6. The identity of charge carriers on the surfaces in the liquid-solid CE. (a) There are both electrons and positive ions on the charged surface in the CE between MgO and DI water. (b) There are both holes and negative ions on the charged surface in the CE between AlN and DI water.

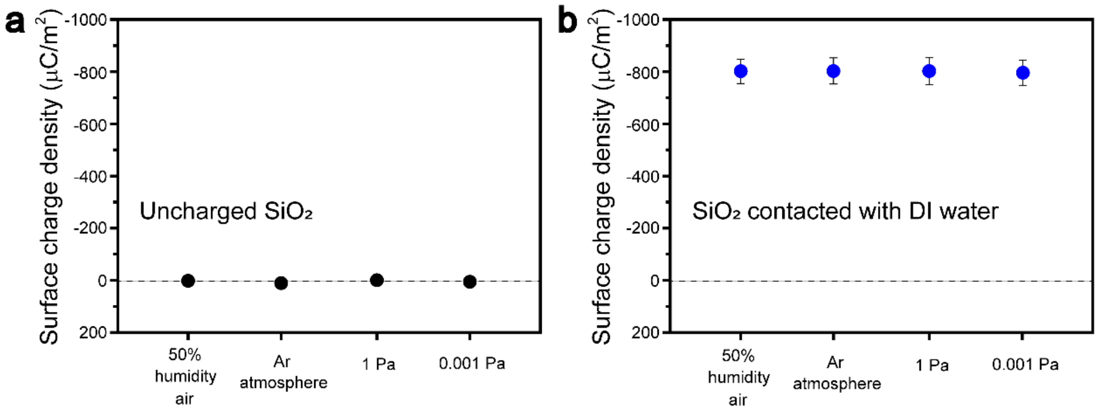

Supplementary Figure 7. The charge density on the SiO_2 surfaces in different atmosphere. (a) before and (b) after contacted with DI water. (AM-KPFM cannot work in vacuum; hence, the FM-KPFM is used here). (Error bar are defined as s. d.).

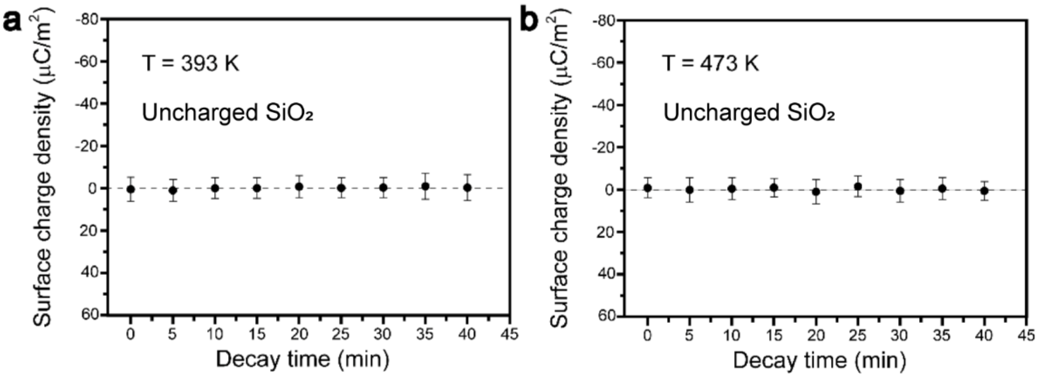

Supplementary Figure 8. The change of charge density on the uncharged SiO_2 surfaces. At (a) 393 K and (b) 473 K. (Error bar are defined as s. d.).

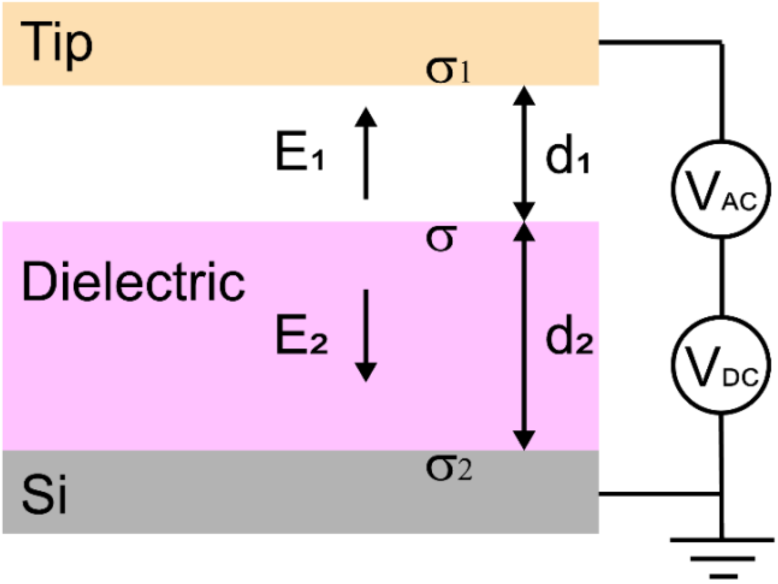

Supplementary Figure 9. The parallel plate capacitor model for KPFM mode.

Supplementary Notes

Supplementary Note 1. The *ab initio* molecular dynamics simulations.

The *ab initio* molecular dynamics was performed to demonstrate the O^- ion will stay on the sample surfaces at 513 K, which is the highest temperature used in this work.

As shown in Supplementary Fig. 1, the initial structure of SiO_2 surface with an O^- ion is established. In the model, the crystal indices of SiO_2 surface are (1 1 1) and the O^- ion is put on the surface. The model was simulated in NVT ensemble, and the temperature was set to be 513 K. The generalized gradient approximation (GGA) as parameterized by Perdew–Burke–Ernzerhof (PBE) was employed for the exchange correlation energy.[1] The plane-wave kinetic-energy cutoff was set at 340 eV, the convergence energy change was set to 2×10^{-6} eV per atom, and a Monkhorst–Pack k-point 3 × 3 × 2 mesh was used in the simulations. The CASTEP code was used to implement the calculations.[2] And the simulation result is shown in Supplementary Movie 1. It can be seen that O^- ion will stay and oscillate on the SiO_2 surface at 513 K.

The behaviors of ions on other material surfaces, which were used in the experiments, were also simulated. The initial structures of the models are shown in

Supplementary Fig. 2, the crystal indices of Al_2O_3, Si_3N_4, AlN, Ta_2O_5, HfO_2, MgO surface are (0 0 1), (1 1 1), (0 0 1), (0 0 1), (0 0 1) and (1 1 1), respectively. And the O^- ion or H^+ is put on the surfaces according to the polarity of the ions stayed on the surfaces in the experiments. The simulation results are shown in the Supplementary Movies 2-7. It can be seen that O^- ion or H^+ ion will also stay and oscillate on the other sample surfaces at 513 K.

Supplementary Note 2. The surface ionization reactions.

As descripted in previous studies,[3] the ionization reaction between oxide surface and water is caused by the amphoteric surface groups, and the ionization reaction can be expressed as following:

$$\equiv \mathrm{A-OH+OH^-} \Leftrightarrow \equiv \mathrm{A-O^- + H_2O} \quad 1$$

$$\equiv \mathrm{A-OH+H^+} \Leftrightarrow \equiv \mathrm{A-OH_2^+} \quad 2$$

where ‘A’ represents the oxidized atom, such as ‘Si’ in SiO_2, ‘Al’ in Al_2O_3.[4]

For Si_3N_4, there are amine groups ($SiNH_2$) on the surface[5] and adsorb the H^+ ions as shown bellow[6]:

$$\equiv \mathrm{SiNH_2 + H^+} \Leftrightarrow \equiv \mathrm{SiNH_3^+} \quad 3$$

For AlN, it has been demonstrated that water molecules will induce N vacancy on the AlN surface, and the N vacancy will be occupied by the O^{2-}.[7] And then, the Supplementary ionization reactions 1 and 2 will occur since there are Al-O bonds on the AlN surface.

Supplementary Note 3. Effects of adsorbed water layers on surface charge density.

The water adsorption on solid surfaces was found to be persistent to high temperatures and even ultra-high vacuum. Hence, the effect of water layers on the charging is considered here.

As we known, the amount of the water molecules adsorbed on the insulator surface is highly dependent on the atmosphere. The surface charge density of the SiO_2 (both charged and uncharged SiO_2) was measured in different atmospheres by using

our home-made vacuum AFM[8] (50% humidity air, Ar atmosphere, 1 Pa vacuum and 0.001 Pa vacuum), as shown in Supplementary Fig. 7. There were much more water molecules on the SiO_2 surface in 50% humidity air than that in 0.001 Pa vacuum condition (or that in Ar atmosphere). However, the results show that no matter the SiO_2 surface was charged (Supplementary Fig. 7b) or not (Supplementary Fig. 7a), the surface charge density remained unchanged in different atmospheres. It indicates that the adsorption and desorption of the water molecules on SiO_2 surface can not affect the surface charge density significantly.

Supplementary Note 4. Effects of temperature on the measurements.

The blank experiments were performed to exclude the effects of temperature on the measurements. As shown in Supplementary Fig. 8. The charge density of the uncharged SiO_2 surfaces was measured at different temperature for 40 mins (393 K, Supplementary Fig. 8a; 473 K, Supplementary Fig. 8b). The results show that the charge density of the SiO_2 surfaces remained zero at 393 K and 473 K. It indicates that the observed changes in the surface potential in our experiments are not due to the temperature effects on the measurements.

Supplementary Note 5. Working principle of KPFM for surface charge density measurement.

KPFM is usually used to measure the contact potential difference (CPD) between a metal and the conductive tip. The working principle of KPFM for the CPD measurement is shown in somewhere else.[9] When the metal (or conductive material) is covered with an insulating layer, the KPFM potential is not only depended on the CPD between the metal (or conductive material) and the tip, but also the charge density on the insulator surface. Hence, the KPFM can also be used to measure the surface charge density of insulator. As shown in Supplementary Fig. 9, it can be considered as a parallel plate capacitor system when the conductive tip scanning the dielectric layer with a conductive substrate (heavily doped silicon used in our experiments). Assume that the charge density on the dielectric surface is σ, induced

charge density on the tip surface is σ_1, and induced charge density on the conductive silicon surface is σ_2. The electric field in the gap between tip and dielectric surface is E_1, and the electric field in the dielectric is E_2.

According to Gauss theorem, the relation between electric fields and surface densities can be expressed with following equations.

$$E_1 = \frac{\sigma_1}{\varepsilon_0} \quad 4$$

$$E_2 = \frac{\sigma_2}{\varepsilon_0 \varepsilon_d} \quad 5$$

$$\sigma_1 + \sigma_2 + \sigma = 0 \quad 6$$

where ε_0 denotes the vacuum dielectric constant and ε_d denotes the relative dielectric constant of the dielectric layer.

In KPFM mode, a DC bias (V_{DC}) and an AC bias ($V_{AC}\sin(\omega t)$) are applied between the tip and the conductive substrate, hence, the potential difference between the tip and the conductive substrate can be written as below:

$$V = V_{DC} + V_{AC}\sin(\omega t) + V_{CPD} \quad 7$$

where V denotes the potential difference between the tip and the conductive substrate, and V_{CPD} denotes the contact potential difference between the tip and the conductive substrate.

Also, the potential difference between the tip and the conductive substrate can be described as:

$$V = E_1 d_1 - E_2 d_2 \quad 8$$

where d_1 denotes the distance between the tip and dielectric surface, and d_2 denotes the thickness of the dielectric layer, as shown in Supplementary Fig. 9.

Combining Supplementary Equations 4 to 8, the electric field between the tip and dielectric surface can be expressed as following:

$$E_1 = \frac{V\varepsilon_o\varepsilon_d - \sigma d_2}{\varepsilon_o d_2 + \varepsilon_o \varepsilon_d d_1} \quad 9$$

And the electric field force on the tip:

$$F_{tip} = E_1 \sigma_1 M = E_1^2 \varepsilon_0 M \quad 10$$

where M denotes the effective area of the tip.

Combining Supplementary Equations 7, 9 and 10, the electric filed force can be expressed as following:

$$F_{tip} = F_c + F_\omega + F_{2\omega} \quad 11$$

where:

$$F_c = (-2(V_{DC} + V_{CPD})\sigma d_2 \varepsilon_o \varepsilon_d + (\sigma d_2)^2 + (V_{DC} + V_{CPD})^2 (\varepsilon_o \varepsilon_d)^2) \cdot \frac{\varepsilon_0 M}{(\varepsilon_o d_2 + \varepsilon_o \varepsilon_d d_1)^2} \quad 12$$

$$F_\omega = 2V_{AC} \sin(\omega t) \left(V_{DC} + V_{CPD} - \frac{\sigma d_2}{\varepsilon_o \varepsilon_d}\right) (\varepsilon_o \varepsilon_d)^2 \frac{\varepsilon_0 M}{(\varepsilon_o d_2 + \varepsilon_o \varepsilon_d d_1)^2} \quad 13$$

$$F_{2\omega} = V_{AC}^2 \sin^2(\omega t) (\varepsilon_o \varepsilon_d)^2 \frac{\varepsilon_0 M}{(\varepsilon_o d_2 + \varepsilon_o \varepsilon_d d_1)^2} \quad 14$$

In KPFM mode, F_ω is extracted out by the lock-in amplifier, and F_ω is controlled to be zero by adjusting the DC bias (V_{DC}). According to Supplementary Equation 13, it can be obtained that:

$$V_{DC} + V_{CPD} - \frac{\sigma d_2}{\varepsilon_0 \varepsilon_d} = 0 \quad 15$$

Hence,

$$\sigma = \frac{(V_{DC} + V_{CPD})\varepsilon_0 \varepsilon_d}{d_2} \quad 16$$

In Supplementary Equation 16, V_{CPD} between the tip and the conductive substrate was measured by directly scanning the substrate in KPFM mode before the substrate is covered by the insulator, and d_2, ε_0 and ε_d are known. V_{DC} is what we obtain in KPFM measurement, it can be called as "surface potential". And it is applied by the Kelvin controller, hence, it can be also named as "Kelvin potential".

Supplementary References

1. Lin, Z. & Bristowe, P. D. Microscopic characteristics of the Ag(111)/ZnO(0001) interface present in optical coatings. *Phys. Rev. B: Condens. Matter Mater. Phys.* **75**, 205423 (2007).
2. Clark, S. J., Segall, M. D., Pickard, C. J., Hasnip, P. J., Probert, M. J., Refson, K. & Payne, M. C. First-principle methods using CASTEP. *Z. Kristallogr.* **220**,

567-570 (2005).

3. S. Usui, Electrical double-layer interaction between oppositely charged dissimilar oxide surfaces with charge regulation and Stern-Grahame layers. *J. Coll. Inter. Sci*. **320**, 353-359 (2008).
4. Bousse, L., Rooij, N. & Bergveld, P. Operation of chemically sensitive field-effect sensors as a function of the insulator-electrolyte interface. *IEEE T. Electron Dev*. **30**, 1263-1270 (1983).
5. Raiteri, R., Martinoia, S. & Grattarola, M. pH-dependent charge density at the insulator-electrolyte interface probed by a scanning force microscope. *Biosens. Bioelectron*. **11**, 1009-1017 (1995).
6. Galassi, C. & Bertoni, F. Water-based Si3N4 suspensions: Part I. Effect of processing routes on the surface chemistry and particle interactions. *J. Mater. Res*. **15**, 155-163 (2000).
7. Chen, Y., Hou, X., Fang, Z. & Wang, E. J. Chen, G. Bei, Adsorption and reaction of water on the AlN (0001) surface from first principles. *J. Phys. Chem. C* **123**, 5460-5468 (2019).
8. Lin, S., Xu, L., Tang, W., Chen, X. & Wang, Z. L. Electron transfer in nano-scale contact electrification: atmosphere effect on the surface states of dielectrics. *Nano Energy*. **65**, 103956 (2019).
9. Melitz, W., Shen, J., Kummel, A. C. & Lee, S. Kelvin probe force microscopy and its application. *Surf. Sci. Rep*. **66**, 1-27 (2011).
