# Supplementary material for: Quantifying electron-transfer in liquid-solid contact electrification and the formation of electric double-layer
Source: Nat Commun. 2020 Jan 21;11:399. doi: 10.1038/s41467-019-14278-9 (PMC6972942; doi:10.1038/s41467-019-14278-9)
Supplement: Supplementary file 3 — Description of Additional Supplementary Files [file 41467_2019_14278_MOESM3_ESM.pdf]

Description of Additional Supplementary Files

File Name: Supplementary Movie 1

Description: The ab initio molecular dynamics simulation result for the behaviors of an O^{-} ions on SiO_2 surface at 513 K.

File Name: Supplementary Movie 2

Description: The ab initio molecular dynamics simulation result for the behaviors of an O^{-} ions on Al_2O_3 surface at 513 K.

File Name: Supplementary Movie 3

Description: The ab initio molecular dynamics simulation result for the behaviors of an H^{+} ions on Si_3N_4 surface at 513 K.

File Name: Supplementary Movie 4

Description: The ab initio molecular dynamics simulation result for the behaviors of an O^{-} ions on AlN surface at 513 K.

File Name: Supplementary Movie 5

Description: The ab initio molecular dynamics simulation result for the behaviors of an H^{+} ions on Ta_2O_5 surface at 513 K.

File Name: Supplementary Movie 6

Description: The ab initio molecular dynamics simulation result for the behaviors of an H^{+} ions on HfO_2 surface at 513 K.

File Name: Supplementary Movie 7

Description: The ab initio molecular dynamics simulation result for the behaviors of an H^{+} ions on MgO surface at 513 K.
